# Reclassification of tumor size for solitary HBV-related hepatocellular carcinoma by minimum *p* value method: a large retrospective study

**DOI:** 10.1186/s12957-020-01963-z

**Published:** 2020-07-24

**Authors:** Hongzhi Liu, Yuan Yang, Chuanchun Chen, Lei Wang, Qizhen Huang, Jianxing Zeng, Kongying Lin, Yongyi Zeng, Pengfei Guo, Weiping Zhou, Jingfeng Liu

**Affiliations:** 1 Southeast Big Data Institute of Hepatobiliary Health, Mengchao Hepatobiliary Hospital of Fujian Medical University, Fuzhou, 350025 People’s Republic of China; 2 Department of Hepatobiliary Surgery, Mengchao Hepatobiliary Hospital of Fujian Medical University, Xihong Road 312, Fuzhou, 350025 People’s Republic of China; 3 Department of Hepatobiliary Surgery, Eastern Hepatobiliary Surgery Hospital, Second Military Medical University, Changhai Street 225, Shanghai, 200438 People’s Republic of China; 4 Fuzhou Yixing Big Data Industry Investment Co., Ltd., Fuzhou, 350025 People’s Republic of China

**Keywords:** Hepatocellular carcinoma, Solitary, Tumor size, Prognosis

## Abstract

**Background and objectives:**

Tumor size is one of the most important issues for hepatocellular carcinoma (HCC) treatment and prognosis, but the classification of it is still controversial. The aim of this study was to screen appropriate cutoffs for size of solitary hepatitis B virus (HBV)-related HCC.

**Methods:**

A cohort of 1760 patients with solitary HBV-related HCC undergoing curative liver resection was divided into 11 groups based on tumor size in 1-cm interval. The minimum *p* value method was used to screen the appropriate size cutoff according to overall survival (OS). If multiple cutoffs meet the above standard, a univariate analysis will be performed by using the Cox proportional hazards regression model, and hazard ratio (HR) will be considered as a criterion to assess the difference in survival.

**Results:**

There are 8 dichotomy, 8 trichotomy, and no inquartation cutoffs that were screened when classifying tumor sizes in accordance with OS. The HR values of tumor size at these trichotomy cutoffs for OS were compared, and the highest HR value is 2.79 when size cutoff is 3/9 cm. Then, we reclassified patients into three new classifications: ≤ 3 cm (*n* = 422), > 3 and ≤ 9 cm (*n* = 1072), and > 9 cm (*n* = 266). The comparison of clinicopathologic characteristics among these three classifications showed that the increase of tumor size was associated with the increase of α-fetoprotein (AFP), microvascular invasion (MVI), tumor differentiation, and liver cirrhosis. And the comparison of the OS among three classifications showed statistical differences.

**Conclusions:**

This study suggested that size criteria of 3 cm and 9 cm in solitary HBV-related HCC patients were appropriate based on biological characteristics and prognostic significance.

## Introduction

Hepatocellular carcinoma (HCC) is the second leading cause of cancer-related death in the world [1, 2]. Due to the high prevalence of hepatitis B virus (HBV) infection, the incidence of HCC in China alone accounts for about 55% cases globally [3]. Currently, liver resection (LR) is regarded as first-line treatment for HCC. Unfortunately, outcomes after resection are still suboptimal [4, 5]. Tumor size has been taken into account to be a poor-prognosis factor for HCC after LR, and it has been included in multiple HCC staging systems. However, there is no consensus regarding the cutoff criteria of size for the solitary HCC at present.

Many previous studies tend to focus on the cutoff of size for small HCC tumor alone or single large HCC tumor which is > 5 cm [6–9]. Some studies analyzed solitary HCC of full size and screened size cutoffs based on overall survival (OS) [10–12]. However, the tumor size cutoff results are inconsistent. In addition, lots of studies showed tumor size of 2 cm, 3 cm, and 5 cm could be the criterion of small HCC and many studies showed tumor size of 5 cm, 7 cm, 8 cm, and 10 cm could be independent predictors of death after LR [10]. These findings call for appropriate methods to distinguish the tumor size of solitary HCC.

The method of minimum *p* value can be used to distinguish the quantitative indexes by the prognostic outcome [13, 14], and it has been used to classify tumor sizes in a multicenter study [12]. The present study employed the minimum *p* value method to investigate whether patients with solitary HBV-related HCC of different sizes differed significantly in OS after LR, and screen appropriate cutoffs of size for solitary HBV-related HCC.

## Patients and methods

### Study cohort

This study was conducted under the guideline of the 1975 Declaration of Helsinki and was approved by the Institutional Ethics Committee of the Mengchao Hepatobiliary Hospital of Fujian Medical University. Informed consent obtained from all patients was written before LR operation. Medical records of HCC patients from June 2008 to December 2014 were extracted from primary liver cancer big data (PLCBD) [15]. Data were extracted by an engineer and were verified by five researchers in this study.

The inclusion criteria were as follows: (1) solitary HCC tumor, (2) Child-Pugh A or B liver function, (3) seropositive for HBV surface antigen (HBsAg) and seronegative for hepatitis C virus antibody (HCV-Ab), and (4) underwent curative hepatectomy. Exclusion criteria were as follows: (1) having received any preoperative anticancer treatments; (2) had a history of other cancers; (3) HCC caused by other reasons such as hepatitis C virus (HCV), alcohol consumption, and cryptogenic disease; and (4) pathological and clinical data are incomplete. Finally, 1760 patients were selected as the study cohort.

### Study design

To examine possible subclassification of solitary HCC, HCCs with the largest tumor diameters ≤ 10 cm were divided into ten groups with 1-cm intervals, and HCCs with the largest tumor diameters > 10 cm were selected as one group. There were 10, 45, and 120 cutoffs of size when classifying our patients into dichotomy, trichotomy, and inquartation groups respectively. The rationale for adopting the appropriate cutoff value for solitary HBV-related HCC was confirmed by the minimum *p* value approach to predict OS after LR.

The OS rates were generated by using the Kaplan-Meier method, and the differences were compared by log-rank test. There was one *p* value when comparing OS of tumor size dichotomy, and the threshold of minimum *p* value was set at *p* < 0.05, *p* < 0.01, *p* < 0.001, or *p* < 0.0001. There were three and six *p* values when comparing pairwise tumor size trichotomy and inquartation. To control multiplicity in multiple comparison, Bonferroni analysis is used for controlling the incidence of type I errors. Hence, the threshold of minimum *p* value was set at *p* < 0.0167, *p* < 0.01, *p* < 0.001, or *p* < 0.0001 when selecting appropriate trichotomy cutoff and *p* < 0.0083, *p* < 0.001, or *p* < 0.0001 when selecting appropriate inquartation cutoff.

If multiple cutoffs meet the above standard, univariate analyses will be performed by using the Cox proportional hazards regression model, and hazard ratio (HR) will be considered as a criterion to assess the difference in survival. In contrast to the Kaplan-Meier method, Cox proportional hazards regression can provide an effect estimate by quantifying the difference in survival between tumor size groups. There was one HR value when comparing OS of tumor size dichotomy, and the highest one was screened according to the previous study [12]. A dummy variable was created when comparing OS of tumor size trichotomy and inquartation, and there existed two and three HR values, respectively. The highest last HR values (> last cutoff vs ≤ the first one) were screened.

### Clinicopathologic variables

The tumor size was based on the largest dimension of the tumor in the resected specimen. Microvascular invasion (MVI) was defined as the presence of tumor cell clusters within the blood vessels lined by the endothelium including the branch of the portal vein, hepatic vein, or capsular vessel [16]. Tumor differentiation was assessed according to the Edmondson-Steiner grade. Tumor stage was determined according to the Barcelona Clinic Liver Cancer (BCLC) staging system and American Joint Committee on Cancer (AJCC) staging system (8th edition) [17, 18].

### Follow-up

Patients were followed up by the serum levels of α-fetoprotein (AFP), ultrasonography, and computed tomography/magnetic resonance imaging at 1 month after LR operation, then every 2 months in the first 6 months, and every 3 months at a later time. The OS was defined as the time interval between the day of the operation and death. The last follow-up data were collected until December 31, 2018.

### Statistical analysis

Continuous values were expressed as mean ± standard deviation (SD) or as median (range) and compared by using the *t* test or Mann-Whitney *U* test. Categorical variables were expressed as number (%) and compared by using the chi-square or Fisher’s exact test. The OS rates were generated by using the Kaplan-Meier method, and the differences were compared by log-rank test. Univariate analyses were performed by employing the Cox proportional hazards regression model. Statistical analyses were performed by using IBM SPSS software (version 19.0, SPSS Inc., Chicago, IL). A *p* < 0.05 (two-tailed) was considered as the threshold of significance.

## Results

### Clinicopathologic characteristics

The clinicopathologic characteristics of the 1760 solitary HBV-related HCC patients are summarized in Table 1. Most of these patients were male (85.1%), and the mean age was 50.7 (SD 10.2). Over half of the patients (54.5%) were detected with a positive HBVDNA (> 1000 IU/mL). Half of the patients (47.3%) were diagnosis of cirrhosis pathologically. There are only 583 (33.1%) who had AFP levels ≥ 400 ng/mL, and MVI incidence is 23.1%. The mean tumor diameter was 5.55 cm.

**Table 1:** The clinicopathologic factors of solitary HBV-related HCC patients who underwent initial hepatectomy (*n* = 1760)

Parameter	Value
Age, years	50.7 ± 10.2
Sex	
Male	1497 (85.1%)
Female	263 (14.9%)
α-Fetoprotein, ng/mL	
≤ 20	726 (41.2%)
20–400	451 (25.6%)
≥ 400	583 (33.1%)
HBV DNA, IU/ml	
≤ 1000	801 (45.5%)
> 1000	959 (54.5%)
White blood cells, 10^9^/L	5.26 ± 1.74
Platelet count, 10^9^/L	152 (23-479)
Albumin, g/L	41.7 ± 3.74
Total bilirubin, μmol/L	13.4 (3.3–45.4)
γ-Glutamyl transferase, IU/L	55.0 (10–1175)
Alkaline phosphatase, IU/L	80.0 (17–1155)
ALBI grade	
≤ − 2.63	1270 (72.2%)
> 2.63	490 (27.8%)
Blood loss, mL	
< 800	1650 (93.8%)
≥ 800	110 (6.2%)
Tumor size, cm	5.55 ± 3.48
Differentiation degree	
I/II	295 (16.8%)
III/IV	1465 (83.2%)
Microvascular invasion	
Absent	1354 (76.9%)
Present	406 (23.1%)
AJCC stage	
T1a	121 (6.9%)
T1b	1250 (71.0%)
T2	389 (22.1%)
BCLC stage	
0	121 (6.9%)
A	1639 (93.1%)
Tumor capsule	
Incomplete/absent	1232 (70.0%)
Complete	528 (30.0%)
Cirrhosis	
Absent	927 (52.7%)
Present	833 (47.3%)

Values shown are mean ± SD, median (range), or *n* (%)

### Tumor overall survival outcomes

**Fig. 1:**
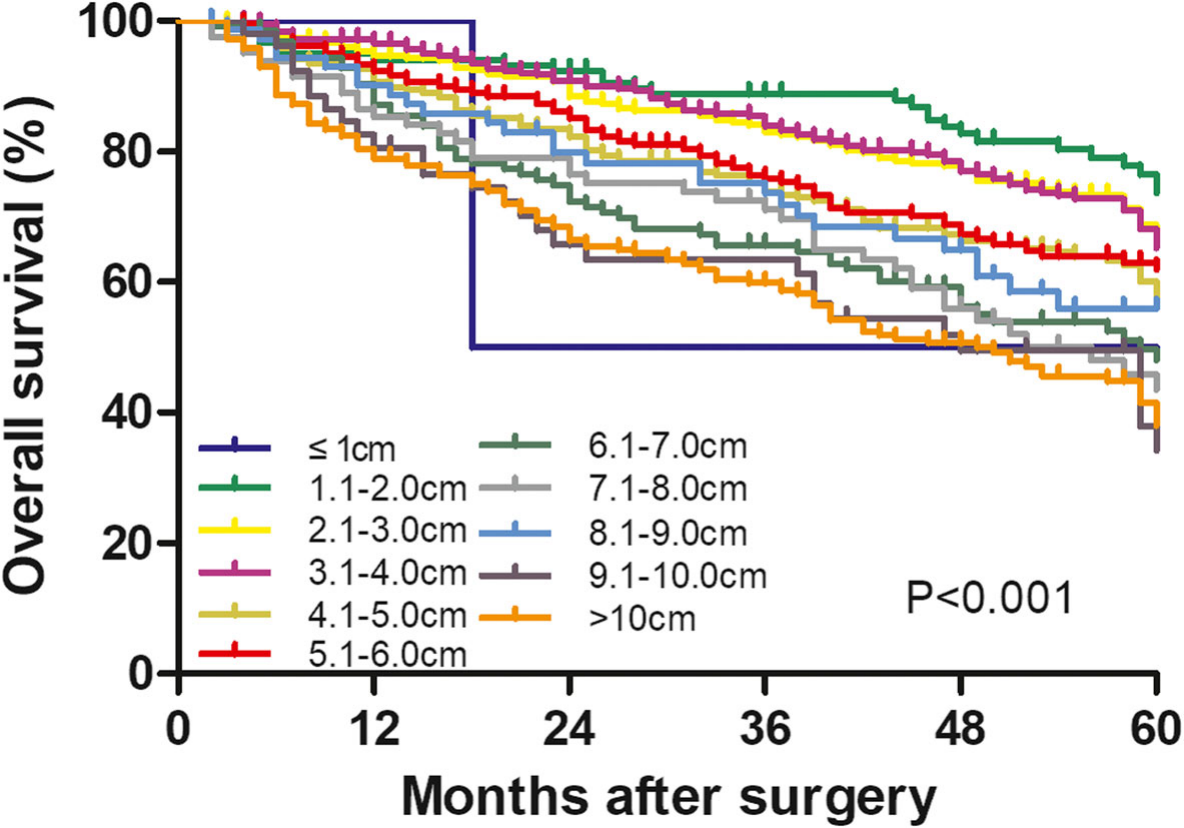
Overall survival of patients with solitary HBV-related HCC following liver resection, classified by tumor size into 11 groups differing at 1.0-cm intervals

Among all 1760 patients, the median survival time was 70 months. The 1-, 3-, and 5-year OS were 90.7%, 75.9%, and 57.8%, respectively. Patients were classified into 11 groups based on tumor size with 1-cm intervals, and the OS decreased with increasing tumor size (Fig. 1).

### Tumor size cutoffs screening based on minimum *p* value of prognosis

To examine possible cutoffs of solitary HCC, the largest tumor diameters were subdivided by 10 dichotomy, 45 trichotomy, and 120 inquartation groups, and there were 10, 45, and 120 cutoffs after exhaustive search. The *p* values of log-rank test for OS of each cutoff were calculated and cutoffs were screened.

As much as 8 from 10 cutoffs were screened when classified tumor diameters by dichotomy, and the minimum *p* value was set at *p* < 0.0001. The HR values of tumor size at these cutoffs for OS were compared, and the highest is 2.074 when size cutoff is 9 cm (Table 2). Similarly, there were 8 from 45 cutoffs which were screened when classifying tumor size by trichotomy, and the minimum *p* value was set at *p* < 0.0001 (Table 3). The HR values of tumor size at these cutoffs for OS were compared, and the highest last HR value is 2.79 when size cutoff is 3/9 cm. However, there was no inquartation cutoff screened when the minimum *p* value was set at *p* < 0.0083.

**Table 2:** The *p* values of log-rank test and HR values for OS of each dichotomy size cutoff

Cutoffs	Log-rank OS	Cox
	*p* value	HR-OS (95% CI)
1 cm	0.40435	
2 cm	0.00016	
3 cm*	< 0.0001	1.702 (1.408–2.059)
4 cm*	< 0.0001	1.881 (1.613–2.194)
5 cm*	< 0.0001	1.87 (1.615–2.164)
6 cm*	< 0.0001	2.049 (1.766–2.377)
7 cm*	< 0.0001	1.968 (1.683–2.302)
8 cm*	< 0.0001	1.948 (1.650–2.299)
9 cm*	< 0.0001	2.074^#^ (1.737–2.476)
10 cm*	< 0.0001	2.013 (1.662–2.439)

*OS* overall survival, *HR* hazard ratio, *CI* confidence interval

*The *p* values of log-rank test for OS of size cutoff had significant statistical difference (*p* < 0.0001); ^#^ The highest HR value of Cox proportional analysis for OS

**Table 3:** The *p* values of log-rank test and HR values for OS of each trichotomy size cutoff

Cutoffs (cm)		*p* value of log-rank			HR (95% CI) of Cox	
A	B	≤ A vs. A~B	≤ A vs. > B	A~B vs. > B	A~B vs. ≤ A	> B vs. ≤ A
1	2	0.611280	0.417322	0.000224		
1	3	0.401394	0.424487	< 0.0001		
1	4	0.588828	0.327959	< 0.0001		
1	5	0.566227	0.277350	< 0.0001		
1	6	0.545619	0.222792	< 0.0001		
1	7	0.493309	0.263552	< 0.0001		
1	8	0.482870	0.257788	< 0.0001		
1	9	0.460243	0.289755	< 0.0001		
1	10	0.444595	0.325466	< 0.0001		
2	3	0.083743	< 0.0001	< 0.0001		
2	4	0.103040	< 0.0001	< 0.0001		
2	5	0.029683	< 0.0001	< 0.0001		
2	6	0.018186	< 0.0001	< 0.0001		
2	7	0.005548	< 0.0001	< 0.0001		
2	8	0.002914	< 0.0001	< 0.0001		
2	9	0.001960	< 0.0001	< 0.0001		
2	10	0.001138	< 0.0001	< 0.0001		
3	4	0.451643	< 0.0001	< 0.0001		
3	5	0.028445	< 0.0001	< 0.0001		
3	6	0.009799	< 0.0001	< 0.0001		
3	7	0.000557	< 0.0001	< 0.0001		
3	8*	< 0.0001	< 0.0001	< 0.0001	1.472 (1.208–1.793)	2.574 (2.058–3.219)
3	9*	< 0.0001	< 0.0001	< 0.0001	1.497 (1.23–1.82)	2.79^#^ (2.21–3.523)
3	10*	< 0.0001	< 0.0001	< 0.0001	1.542 (1.27–1.874)	2.781 (2.176–3.554)
4	5	0.000843	< 0.0001	0.001334		
4	6	0.000214	< 0.0001	< 0.0001		
4	7*	< 0.0001	< 0.0001	< 0.0001	1.566 (1.312–1.868)	2.405 (2.009–2.879)
4	8*	< 0.0001	< 0.0001	< 0.0001	1.63 (1.376–1.932)	2.464 (2.038–2.978)
4	9*	< 0.0001	< 0.0001	< 0.0001	1.646 (1.395–1.944)	2.67 (2.184–3.263)
4	10*	< 0.0001	< 0.0001	< 0.0001	1.702 (1.447–2.004)	2.662 (2.148–3.299)
5	6	0.074289	< 0.0001	< 0.0001		
5	7	< 0.0001	< 0.0001	0.000524		
5	8	< 0.0001	< 0.0001	0.001047		
5	9*	< 0.0001	< 0.0001	< 0.0001	1.602 (1.352–1.9)	2.42 (2.004–2.922)
5	10	< 0.0001	< 0.0001	0.000794		
6	7	< 0.0001	< 0.0001	0.381256		
6	8	< 0.0001	< 0.0001	0.255359		
6	9	< 0.0001	< 0.0001	0.025411		
6	10	< 0.0001	< 0.0001	0.069108		
7	8	0.000275	< 0.0001	0.449406		
7	9	< 0.0001	< 0.0001	0.037663		
7	10	< 0.0001	< 0.0001	0.109361		
8	9	0.044308	< 0.0001	0.042670		
8	10	< 0.0001	< 0.0001	0.166143		
9	10	< 0.0001	< 0.0001	0.944208		

*OS* overall survival, *HR* hazard ratio, *CI* confidence interval

*The *p* values of log-rank test for OS of size cutoff had significant statistical difference (*p* < 0.0001); ^#^ The highest HR value of Cox proportional analysis for OS

### Redefinition of tumor size groups and their characteristics

We reclassified patients into three new categories: ≤ 3 cm (*n* = 422), > 3 and ≤ 9 cm (*n* = 1072), and > 9 cm (*n* = 266). The comparison of clinicopathologic characteristics among these three new classifications (Table 4) showed that the increase of tumor size was associated with the increase of AFP, white blood cell count (WBC), platelet count (PLT), γ-glutamyl transferase (GGT), alkaline phosphatase (ALP), volume of blood loss, MVI, tumor differentiation, and liver cirrhosis.

**Table 4:** Comparison of clinicopathologic characteristics among three new classifications

Parameter	≤ 3 cm (*n* = 422)	3~9 cm (*n* = 1072)	> 9 cm (*n* = 266)	*p* value		
				≤ 3 vs. 3~9	3~9 vs. > 9	≤ 3 vs. > 9
Age, years						
≤ 50	212 (50.2%)	534 (49.8%)	139 (52.3%)	0.928	0.519	0.662
> 50	210 (49.8%)	538 (50.2%)	127 (47.7%)			
Sex						
Male	346 (82.0%)	928 (86.6%)	223 (83.8%)	0.0303	0.293	0.604
Female	76 (18.0%)	144 (13.4%)	43 (16.2%)			
AFP, ng/mL						
≤ 20	183 (43.4%)	471 (43.9%)	72 (27.1%)	< 0.001	< 0.001	< 0.001
20–400	140 (33.2%)	249 (23.2%)	62 (23.3%)			
≥ 400	99 (23.5%)	352 (32.8%)	132 (49.6%)			
HBV DNA, IU/ml						
≤ 1000	213 (50.5%)	488 (45.5%)	100 (37.6%)	0.0951	0.0236	0.00126
> 1000	209 (49.5%)	584 (54.5%)	166 (62.4%)			
White blood cells, 10^9^/L						
< 4	131 (31.0%)	236 (22.0%)	41 (15.4%)	< 0.001	0.0218	< 0.001
≥ 4	291 (69.0%)	836 (78.0%)	225 (84.6%)			
Platelet count, 10^9^/L						
< 100	115 (27.3%)	193 (18.0%)	15 (5.6%)	< 0.001	< 0.001	< 0.001
≥ 100	307 (72.7%)	879 (82.0%)	251 (94.4%)			
Albumin, g/L						
< 35	10 (2.4%)	29 (2.7%)	14 (5.3%)	0.852	0.0545	0.0717
≥ 35	412 (97.6%)	1043 (97.3%)	252 (94.7%)			
Total bilirubin, μmol/L						
≤ 17.1	314 (74.4%)	822 (76.7%)	205 (77.1%)	0.391	0.958	0.485
> 17.1	108 (25.6%)	250 (23.3%)	61 (22.9%)			
γ-Glutamyl transferase, IU/L						
≤ 64	300 (71.1%)	663 (61.8%)	62 (23.3%)	< 0.001	< 0.001	< 0.001
> 64	122 (28.9%)	409 (38.2%)	204 (76.7%)			
Alkaline phosphatase, IU/L						
≤ 129	410 (97.2%)	998 (93.1%)	181 (68.0%)	0.00362	< 0.001	< 0.001
> 129	12 (2.8%)	74 (6.9%)	85 (32.0%)			
ALBI grade						
≤ − 2.63	321 (76.1%)	795 (74.2%)	154 (57.9%)	0.486	< 0.001	< 0.001
> 2.63	101 (23.9%)	277 (25.8%)	112 (42.1%)			
Blood loss, mL						
< 800	417 (98.8%)	1024 (95.5%)	209 (78.6%)	0.00326	< 0.001	< 0.001
≥ 800	5 (1.2%)	48 (4.5%)	57 (21.4%)			
Differentiation degree						
I/II	105 (24.9%)	174 (16.2%)	16 (6.0%)	<0.001	<0.001	<0.001
III/IV	317 (75.1%)	898 (83.8%)	250 (94.0%)			
Microvascular invasion						
Negative	349 (82.7%)	830 (77.4%)	175 (65.8%)	0.0292	< 0.001	< 0.001
Positive	73 (17.3%)	242 (22.6%)	91 (34.2%)			
Tumor capsule						
Incomplete/absent	292 (69.2%)	735 (68.6%)	205 (77.1%)	0.861	0.00827	0.0309
Complete	130 (30.8%)	337 (31.4%)	61 (22.9%)			
Cirrhosis						
Absent	157 (37.2%)	588 (54.9%)	182 (68.4%)	< 0.001	< 0.001	< 0.001
Present	265 (62.8%)	484 (45.1%)	84 (31.6%)			

### Overall survival of the new three groups

**Fig. 2:**
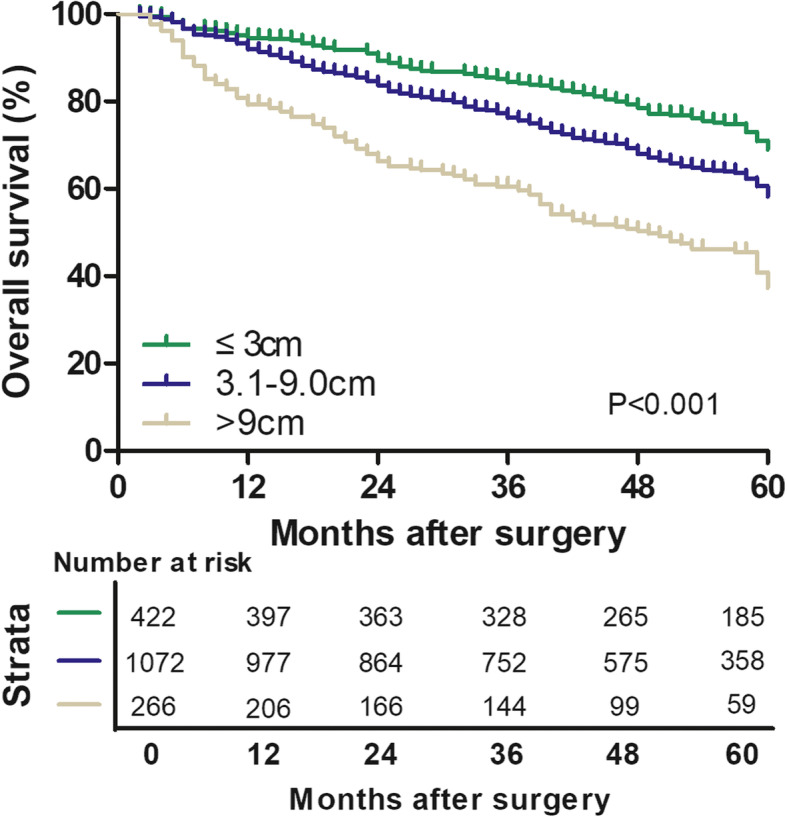
Overall survival of patients with solitary HBV-related HCC classified by tumor size into three new groups

Based on these biological characteristics and prognostic findings, we suggested 3/9 cm could be appropriate cutoff for tumor size of solitary HBV-related HCC. The overall 1-, 3-, and 5-year survival rates were 94.5%, 84.5%, and 69.0% in patients with HCC ≤ 3 cm; 92.0%, 76.3%, and 58.3% in patients with HCC > 3 and ≤ 9 cm; and 79.3%, 60.5%, and 37.4% in patients with HCC > 9 cm, respectively. The comparison of the OS between any two subgroups showed statistical differences (all *p* < 0.001) (Fig. 2).

## Discussion

Tumor size is a key characteristic of HCC, and its classification is one of the most important issues for HCC treatment and prognosis. However, there are currently no uniform cutoff criteria for the size of HCC. A systematic review showed there are three kinds of criteria for small HCC alone [19]. Even in different stage systems, the criteria for the size of solitary HCC are inconsistent. In this study, we subdivided tumor size by 1-cm intervals and analyzed the relationship between tumor size and the overall survival of 1760 patients with solitary HBV-related HCC. Our results revealed a stepwise incremental deterioration in OS outcomes with increased tumor size, which is consistent with other studies [10, 11]. Then we employed the minimum *p* value method to screen the appropriate size cutoffs that could divide HCC tumor OS well.

Many previous studies showed that multiple dichotomy can divide tumor size well based on prognosis. The BCLC, AJCC, and Japan Integrated Staging Score (JIS) employ 2 cm as the cutoff for single HCC [17, 18, 20], while the Hong Kong Liver Cancer (HKLC) and Chinese Liver Cancer (CNLC) staging system use a cutoff with 5 cm [21, 22]. One previous study suggested multiple size cutoffs such as 2 cm, 3 cm, 4 cm, 5 cm, 8 cm, and 10 cm have good discrimination for HCC prognosis [23]. In this study, similarly, dichotomy results of minimum *p* value of OS showed that 8 among 10 cutoffs can divide tumor size into 2 groups well. These evidences indicated dichotomy of HCC tumor size might not reflect the biological nature of HCC. Among these cutoffs, the HR values of OS are compared, and 9 cm has the highest HR for OS. This is inconsistent with the result of a multicenter study indicating 2 cm has the highest HR for OS [12], which may be caused by different study population and treatment decisions in different countries or regions.

Then, we divided HCC tumors into 45 trichotomy groups. There are 8 cutoffs of size discriminated OS well when the minimum *p* value was set at *p* < 0.0001. With the comparison of HR of these groups, we found that 3/9 cm cutoff groups have the highest HR value for OS. When we tried inquartation of tumor size, there were no cutoffs screened. Furthermore, the comparison of clinicopathologic characteristics of new classifications showed that the increase of tumor size was associated with biological characteristics. On the basis of these results, 3/9 cm could be an appropriate size cutoff for HCC tumor.

There are some similarities and differences with other studies. In one study of 857 patients with single HCC, 5/8 cm was suggested to be the cutoff of size after dividing HCC into 5 groups with 2-cm intervals and combining the adjacent groups with similar OS [10]. Another large retrospective study chose the controversial cutoff of 5 cm as the boundary of small and large HCC after a similar method [11]. As the initial criteria of small HCC, 5 cm was raised since the mid to late 1970s [24]. Along with the advances in radiographic diagnostic techniques and pathophysiology, smaller criteria such as 3 cm and 2 cm were proposed to replace the criteria of small HCC by many East-West study groups. The size cutoff of 2 cm was raised from BCLC system in 2003 based on the data of the Liver Cancer Study Group of Japan (LCSGJ) and has been adopted in the BCLC and AJCC staging system (eight edition). However, many studies showed tumors up to 2 cm are accounted for a very small proportion of HCC and hard to analyze their pathobiological characteristics [25]. Moreover, studies based on pathobiological characteristics indicated that 3 cm in diameter is an important turning point of HCC development, where HCC transformed from relatively benign behavior to a more aggressive progression [6]. From a clinical standpoint, single HCC tumors up to 3 cm had a similar 3-year OS rate when treated by radiofrequency ablation (RFA), percutaneous ethanol injection (PEI), and surgical resection [26]. Thus, 3 cm as a cutoff of small HCC had a pathobiological and treatment significance.

With the development of research, the cutoff of large HCC tumor size was no longer confined to 5 cm, which can be reflected in changes in criteria for liver transplantation. University of California, San Francisco (UCSF) criteria [27] and Up-to-Seven criteria [28] implied single tumors ≤6.5 or ≤ 6 cm had a same prognosis with Milan criteria. Hangzhou criteria [29] and Fudan criteria [30] further broadened the size cutoff of single HCC tumor to 8 cm and 9 cm. In this study, we reclassified patients into three new classifications: ≤ 3 cm, > 3 and ≤ 9 cm, and > 9 cm according to the results of the minimum *p* value of OS. The comparison of clinicopathologic characteristics among these three groups showed that the increase of tumor size was associated with multiple pathobiological features such as AFP, MVI, tumor differentiation, and liver cirrhosis. In addition, the comparison of the overall survival between any two subgroups showed a statistical difference (all *p* < 0.001). These indicated that 3/9 cm as the boundary of small HCC and large HCC had a biological meaning and prognostic significance.

There are a few limitations to this study. Firstly, although the study population is large enough, this is a retrospective study and thus the results may not be generalized. A multicenter prospective study may be necessary to perform to validate our results. Secondly, all of the study population was HBV-related HCC since their characteristics are different from non-HBV-related HCC. Thirdly, insufficient patient volume of HCC ≤ 1 cm may lead to be hard to work out further subclassification of HCC tumor size.

In conclusion, this study suggested that the tumor size with a cutoff of 3 cm and 9 cm in solitary HBV-related HCC patients was appropriate based on biological characteristics and prognostic significance.

## Supplementary information

**Additional file 1: Table Supplement. Table S1.** The p values of log-rank test and HR values for RFS of each dichotomy size cutoff. **Table S2.** The p values of log-rank test and HR values for RFS of each trichotomy size cutoff. **Table S3.** The p values of log-rank test for RFS of each inquartation size cutoff.

## Data Availability

All data generated or analyzed during this study are included in the articles.
