# Supplementary material for: Reclassification of tumor size for solitary HBV-related hepatocellular carcinoma by minimum p value method: a large retrospective study
Source: World J Surg Oncol. 2020 Jul 24;18:185. doi: 10.1186/s12957-020-01963-z (PMC7382134; doi:10.1186/s12957-020-01963-z)
Supplement: Supplementary file 1 — Additional file 1: Table Supplement. Table S1. The p values of log-rank test and HR values for RFS of each dichotomy size cutoff. Table S2. The p values of log-rank test and HR values for RFS of each trichotomy size cutoff. Table S3. The p values of log-rank test for RFS of each inquartation size cutoff. [file 12957_2020_1963_MOESM1_ESM.docx]

**Table Supplement**

Table S1. The p values of log-rank test and HR values for RFS of each dichotomy size cutoff

| Cutoffs | Log-rank OS | Cox |
| --- | --- | --- |
|  | *P*-value | HR-OS (95%CI) |
| 1cm | 0.98712 | 0.984 (0.138-6.994) |
| 2cm | 0.00133 | 1.656 (1.209-2.267) |
| 3cm* | <0.0001 | 1.481 (1.248-1.758) |
| 4cm* | <0.0001 | 1.445 (1.256-1.663) |
| 5cm* | <0.0001 | 1.483 (1.292-1.702) |
| 6cm* | <0.0001 | 1.625 (1.408-1.874) |
| 7cm* | <0.0001 | 1.611 (1.383-1.878) |
| 8cm* | <0.0001 | 1.699 (1.445-1.998) |
| 9cm* | <0.0001 | 1.821 (1.529-2.169) |
| 10cm* | <0.0001 | 1.885 (1.56-2.277) |

*means the p values of log-rank test for OS of size cutoff were significant statistical difference (p<0.0001).

OS, overall survival; HR, hazard ratio; CI, confidence interval.

Table S2. The p values of log-rank test and HR values for RFS of each trichotomy size cutoff

| Cutoffs (cm) | | *P*-value of Log-rank | | | HR (95%CI) of Cox | |
| --- | --- | --- | --- | --- | --- | --- |
| A | B | A~B vs. **≤**A | ≤A vs. >B | A~B vs. >B | A~B vs. **≤**A | >B vs. **≤**A |
| 1 | 2 | 0.594830 | 0.987601 | 0.001223 |  |  |
| 1 | 3 | 0.745366 | 0.943039 | <0.0001 |  |  |
| 1 | 4 | 0.791495 | 0.873865 | <0.0001 |  |  |
| 1 | 5 | 0.837542 | 0.820542 | <0.0001 |  |  |
| 1 | 6 | 0.849291 | 0.739309 | <0.0001 |  |  |
| 1 | 7 | 0.886298 | 0.724131 | <0.0001 |  |  |
| 1 | 8 | 0.895392 | 0.674094 | <0.0001 |  |  |
| 1 | 9 | 0.903216 | 0.601735 | <0.0001 |  |  |
| 1 | 10 | 0.918342 | 0.580573 | <0.0001 |  |  |
| 2 | 3 | 0.147495 | 0.000429 | 0.000724 |  |  |
| 2 | 4 | 0.047168 | 0.000113 | <0.0001 |  |  |
| 2 | 5 | 0.025142 | <0.0001 | <0.0001 |  |  |
| 2 | 6 | 0.021948 | <0.0001 | <0.0001 |  |  |
| 2 | 7 | 0.011381 | <0.0001 | <0.0001 |  |  |
| 2 | 8 | 0.009250 | <0.0001 | <0.0001 |  |  |
| 2 | 9 | 0.007682 | <0.0001 | <0.0001 |  |  |
| 2 | 10 | 0.005933 | <0.0001 | <0.0001 |  |  |
| 3 | 4 | 0.064604 | <0.0001 | 0.004609 |  |  |
| 3 | 5 | 0.016223 | <0.0001 | 0.000149 |  |  |
| 3 | 6 | 0.012319 | <0.0001 | <0.0001 |  |  |
| 3 | 7 | 0.002281 | <0.0001 | <0.0001 |  |  |
| 3 | 8 | 0.001395 | <0.0001 | <0.0001 |  |  |
| 3 | 9* | 0.000824 | <0.0001 | <0.0001 | 1.339 (1.123-1.598) | 2.252 (1.807-2.805) |
| 3 | 10* | 0.000412 | <0.0001 | <0.0001 | 1.359 (1.141-1.62) | 2.362 (1.873-2.978) |
| 4 | 5 | 0.089504 | <0.0001 | 0.015026 |  |  |
| 4 | 6 | 0.069927 | <0.0001 | <0.0001 |  |  |
| 4 | 7 | 0.005336 | <0.0001 | 0.000134 |  |  |
| 4 | 8 | 0.002597 | <0.0001 | <0.0001 |  |  |
| 4 | 9 | 0.001198 | <0.0001 | <0.0001 |  |  |
| 4 | 10* | 0.000390 | <0.0001 | <0.0001 | 1.298 (1.118-1.508) | 2.149 (1.75-2.639) |
| 5 | 6 | 0.452485 | <0.0001 | 0.001529 |  |  |
| 5 | 7 | 0.016983 | <0.0001 | 0.003778 |  |  |
| 5 | 8 | 0.007114 | <0.0001 | 0.000371 |  |  |
| 5 | 9 | 0.002715 | <0.0001 | <0.0001 |  |  |
| 5 | 10* | 0.000614 | <0.0001 | <0.0001 | 1.297 (1.11-1.516) | 2.056 (1.688-2.504) |
| 6 | 7 | 0.001602 | <0.0001 | 0.341869 |  |  |
| 6 | 8 | 0.001030 | <0.0001 | 0.036482 |  |  |
| 6 | 9 | 0.000401 | <0.0001 | 0.003466 |  |  |
| 6 | 10 | <0.0001 | <0.0001 | 0.001573 |  |  |
| 7 | 8 | 0.191916 | <0.0001 | 0.051089 |  |  |
| 7 | 9 | 0.053292 | <0.0001 | 0.005162 |  |  |
| 7 | 10 | 0.006711 | <0.0001 | 0.002780 |  |  |
| 8 | 9 | 0.145658 | <0.0001 | 0.056641 |  |  |
| 8 | 10 | 0.012859 | <0.0001 | 0.025398 |  |  |
| 9 | 10 | 0.027675 | <0.0001 | 0.199114 |  |  |

*means the p values of log-rank test for OS of size cutoff were significant statistical difference (p<0.001).

OS, overall survival; HR, hazard ratio; CI, confidence interval.

Table S3. The p values of log-rank test of each inquartation size cutoff

| Cutoffs (cm) | | | *P*-value of Log-rank test | | | | | |
| --- | --- | --- | --- | --- | --- | --- | --- | --- |
| A | B | C | ≤A vs. A~B | ≤A vs. B~C | ≤A vs. >C | A~B vs. B~C | A~B vs. >C | B~C vs. >C |
| 1 | 2 | 3 | 0.595 | 0.801 | 0.943 | 0.136 | 0.000 | 0.001 |
| 1 | 2 | 4 | 0.595 | 0.825 | 0.874 | 0.043 | 0.000 | 0.000 |
| 1 | 2 | 5 | 0.595 | 0.867 | 0.821 | 0.023 | 0.000 | 0.000 |
| 1 | 2 | 6 | 0.595 | 0.875 | 0.739 | 0.020 | 0.000 | 0.000 |
| 1 | 2 | 7 | 0.595 | 0.912 | 0.724 | 0.010 | 0.000 | 0.000 |
| 1 | 2 | 8 | 0.595 | 0.920 | 0.674 | 0.008 | 0.000 | 0.000 |
| 1 | 2 | 9 | 0.595 | 0.927 | 0.602 | 0.007 | 0.000 | 0.000 |
| 1 | 2 | 10 | 0.595 | 0.943 | 0.581 | 0.005 | 0.000 | 0.000 |
| 1 | 3 | 4 | 0.745 | 0.845 | 0.874 | 0.063 | 0.000 | 0.005 |
| 1 | 3 | 5 | 0.745 | 0.899 | 0.821 | 0.016 | 0.000 | 0.000 |
| 1 | 3 | 6 | 0.745 | 0.902 | 0.739 | 0.012 | 0.000 | 0.000 |
| 1 | 3 | 7 | 0.745 | 0.947 | 0.724 | 0.002 | 0.000 | 0.000 |
| 1 | 3 | 8 | 0.745 | 0.955 | 0.674 | 0.001 | 0.000 | 0.000 |
| 1 | 3 | 9 | 0.745 | 0.962 | 0.602 | 0.001 | 0.000 | 0.000 |
| 1 | 3 | 10 | 0.745 | 0.979 | 0.581 | 0.000 | 0.000 | 0.000 |
| 1 | 4 | 5 | 0.791 | 0.966 | 0.821 | 0.089 | 0.000 | 0.015 |
| 1 | 4 | 6 | 0.791 | 0.946 | 0.739 | 0.069 | 0.000 | 0.000 |
| 1 | 4 | 7 | 0.791 | 0.993 | 0.724 | 0.005 | 0.000 | 0.000 |
| 1 | 4 | 8 | 0.791 | 0.989 | 0.674 | 0.003 | 0.000 | 0.000 |
| 1 | 4 | 9 | 0.791 | 0.985 | 0.602 | 0.001 | 0.000 | 0.000 |
| 1 | 4 | 10 | 0.791 | 0.964 | 0.581 | 0.000 | 0.000 | 0.000 |
| 1 | 5 | 6 | 0.838 | 0.916 | 0.739 | 0.451 | 0.000 | 0.002 |
| 1 | 5 | 7 | 0.838 | 0.961 | 0.724 | 0.017 | 0.000 | 0.004 |
| 1 | 5 | 8 | 0.838 | 0.960 | 0.674 | 0.007 | 0.000 | 0.000 |
| 1 | 5 | 9 | 0.838 | 0.959 | 0.602 | 0.003 | 0.000 | 0.000 |
| 1 | 5 | 10 | 0.838 | 0.930 | 0.581 | 0.001 | 0.000 | 0.000 |
| 1 | 6 | 7 | 0.849 | 0.794 | 0.724 | 0.002 | 0.000 | 0.342 |
| 1 | 6 | 8 | 0.849 | 0.855 | 0.674 | 0.001 | 0.000 | 0.036 |
| 1 | 6 | 9 | 0.849 | 0.881 | 0.602 | 0.000 | 0.000 | 0.003 |
| 1 | 6 | 10 | 0.849 | 0.850 | 0.581 | 0.000 | 0.000 | 0.002 |
| 1 | 7 | 8 | 0.886 | 0.955 | 0.674 | 0.192 | 0.000 | 0.051 |
| 1 | 7 | 9 | 0.886 | 0.956 | 0.602 | 0.053 | 0.000 | 0.005 |
| 1 | 7 | 10 | 0.886 | 0.886 | 0.581 | 0.007 | 0.000 | 0.003 |
| 1 | 8 | 9 | 0.895 | 0.954 | 0.602 | 0.145 | 0.000 | 0.057 |
| 1 | 8 | 10 | 0.895 | 0.845 | 0.581 | 0.013 | 0.000 | 0.025 |
| 1 | 9 | 10 | 0.903 | 0.697 | 0.581 | 0.028 | 0.000 | 0.199 |
| 2 | 3 | 4 | 0.147 | 0.029 | 0.000 | 0.274 | 0.000 | 0.005 |
| 2 | 3 | 5 | 0.147 | 0.013 | 0.000 | 0.129 | 0.000 | 0.000 |
| 2 | 3 | 6 | 0.147 | 0.013 | 0.000 | 0.112 | 0.000 | 0.000 |
| 2 | 3 | 7 | 0.147 | 0.006 | 0.000 | 0.039 | 0.000 | 0.000 |
| 2 | 3 | 8 | 0.147 | 0.004 | 0.000 | 0.029 | 0.000 | 0.000 |
| 2 | 3 | 9 | 0.147 | 0.004 | 0.000 | 0.020 | 0.000 | 0.000 |
| 2 | 3 | 10 | 0.147 | 0.003 | 0.000 | 0.013 | 0.000 | 0.000 |
| 2 | 4 | 5 | 0.047 | 0.013 | 0.000 | 0.228 | 0.000 | 0.015 |
| 2 | 4 | 6 | 0.047 | 0.013 | 0.000 | 0.213 | 0.000 | 0.000 |
| 2 | 4 | 7 | 0.047 | 0.003 | 0.000 | 0.032 | 0.000 | 0.000 |
| 2 | 4 | 8 | 0.047 | 0.003 | 0.000 | 0.019 | 0.000 | 0.000 |
| 2 | 4 | 9 | 0.047 | 0.002 | 0.000 | 0.011 | 0.000 | 0.000 |
| 2 | 4 | 10 | 0.047 | 0.001 | 0.000 | 0.005 | 0.000 | 0.000 |
| 2 | 5 | 6 | 0.025 | 0.043 | 0.000 | 0.661 | 0.000 | 0.002 |
| 2 | 5 | 7 | 0.025 | 0.003 | 0.000 | 0.048 | 0.000 | 0.004 |
| 2 | 5 | 8 | 0.025 | 0.002 | 0.000 | 0.025 | 0.000 | 0.000 |
| 2 | 5 | 9 | 0.025 | 0.002 | 0.000 | 0.012 | 0.000 | 0.000 |
| 2 | 5 | 10 | 0.025 | 0.001 | 0.000 | 0.004 | 0.000 | 0.000 |
| 2 | 6 | 7 | 0.022 | 0.000 | 0.000 | 0.004 | 0.000 | 0.342 |
| 2 | 6 | 8 | 0.022 | 0.000 | 0.000 | 0.003 | 0.000 | 0.036 |
| 2 | 6 | 9 | 0.022 | 0.000 | 0.000 | 0.001 | 0.000 | 0.003 |
| 2 | 6 | 10 | 0.022 | 0.000 | 0.000 | 0.000 | 0.000 | 0.002 |
| 2 | 7 | 8 | 0.011 | 0.014 | 0.000 | 0.263 | 0.000 | 0.051 |
| 2 | 7 | 9 | 0.011 | 0.004 | 0.000 | 0.094 | 0.000 | 0.005 |
| 2 | 7 | 10 | 0.011 | 0.001 | 0.000 | 0.016 | 0.000 | 0.003 |
| 2 | 8 | 9 | 0.009 | 0.010 | 0.000 | 0.202 | 0.000 | 0.057 |
| 2 | 8 | 10 | 0.009 | 0.001 | 0.000 | 0.025 | 0.000 | 0.025 |
| 2 | 9 | 10 | 0.008 | 0.001 | 0.000 | 0.043 | 0.000 | 0.199 |
| 3 | 4 | 5 | 0.065 | 0.021 | 0.000 | 0.564 | 0.001 | 0.015 |
| 3 | 4 | 6 | 0.065 | 0.014 | 0.000 | 0.627 | 0.000 | 0.000 |
| 3 | 4 | 7 | 0.065 | 0.001 | 0.000 | 0.237 | 0.000 | 0.000 |
| 3 | 4 | 8 | 0.065 | 0.001 | 0.000 | 0.189 | 0.000 | 0.000 |
| 3 | 4 | 9 | 0.065 | 0.000 | 0.000 | 0.147 | 0.000 | 0.000 |
| 3 | 4 | 10 | 0.065 | 0.000 | 0.000 | 0.097 | 0.000 | 0.000 |
| 3 | 5 | 6 | 0.016 | 0.084 | 0.000 | 0.982 | 0.000 | 0.002 |
| 3 | 5 | 7 | 0.016 | 0.001 | 0.000 | 0.196 | 0.000 | 0.004 |
| 3 | 5 | 8 | 0.016 | 0.000 | 0.000 | 0.140 | 0.000 | 0.000 |
| 3 | 5 | 9 | 0.016 | 0.000 | 0.000 | 0.093 | 0.000 | 0.000 |
| 3 | 5 | 10 | 0.016 | 0.000 | 0.000 | 0.045 | 0.000 | 0.000 |
| 3 | 6 | 7 | 0.012 | 0.000 | 0.000 | 0.015 | 0.000 | 0.342 |
| 3 | 6 | 8 | 0.012 | 0.000 | 0.000 | 0.016 | 0.000 | 0.036 |
| 3 | 6 | 9 | 0.012 | 0.000 | 0.000 | 0.011 | 0.000 | 0.003 |
| 3 | 6 | 10 | 0.012 | 0.000 | 0.000 | 0.003 | 0.000 | 0.002 |
| 3 | 7 | 8 | 0.002 | 0.020 | 0.000 | 0.438 | 0.000 | 0.051 |
| 3 | 7 | 9 | 0.002 | 0.002 | 0.000 | 0.230 | 0.000 | 0.005 |
| 3 | 7 | 10 | 0.002 | 0.000 | 0.000 | 0.067 | 0.000 | 0.003 |
| 3 | 8 | 9 | 0.001 | 0.012 | 0.000 | 0.343 | 0.000 | 0.057 |
| 3 | 8 | 10 | 0.001 | 0.000 | 0.000 | 0.073 | 0.000 | 0.025 |
| 3 | 9 | 10 | 0.001 | 0.001 | 0.000 | 0.084 | 0.000 | 0.199 |
| 4 | 5 | 6 | 0.090 | 0.268 | 0.000 | 0.779 | 0.001 | 0.002 |
| 4 | 5 | 7 | 0.090 | 0.006 | 0.000 | 0.454 | 0.001 | 0.004 |
| 4 | 5 | 8 | 0.090 | 0.002 | 0.000 | 0.394 | 0.000 | 0.000 |
| 4 | 5 | 9 | 0.090 | 0.001 | 0.000 | 0.325 | 0.000 | 0.000 |
| 4 | 5 | 10 | 0.090 | 0.000 | 0.000 | 0.227 | 0.000 | 0.000 |
| 4 | 6 | 7 | 0.070 | 0.000 | 0.000 | 0.036 | 0.000 | 0.342 |
| 4 | 6 | 8 | 0.070 | 0.000 | 0.000 | 0.046 | 0.000 | 0.036 |
| 4 | 6 | 9 | 0.070 | 0.000 | 0.000 | 0.039 | 0.000 | 0.003 |
| 4 | 6 | 10 | 0.070 | 0.000 | 0.000 | 0.017 | 0.000 | 0.002 |
| 4 | 7 | 8 | 0.005 | 0.059 | 0.000 | 0.633 | 0.000 | 0.051 |
| 4 | 7 | 9 | 0.005 | 0.009 | 0.000 | 0.418 | 0.000 | 0.005 |
| 4 | 7 | 10 | 0.005 | 0.001 | 0.000 | 0.181 | 0.000 | 0.003 |
| 4 | 8 | 9 | 0.003 | 0.040 | 0.000 | 0.482 | 0.000 | 0.057 |
| 4 | 8 | 10 | 0.003 | 0.001 | 0.000 | 0.157 | 0.000 | 0.025 |
| 4 | 9 | 10 | 0.001 | 0.004 | 0.000 | 0.151 | 0.000 | 0.199 |
| 5 | 6 | 7 | 0.452 | 0.001 | 0.000 | 0.060 | 0.001 | 0.342 |
| 5 | 6 | 8 | 0.452 | 0.001 | 0.000 | 0.080 | 0.000 | 0.036 |
| 5 | 6 | 9 | 0.452 | 0.000 | 0.000 | 0.082 | 0.000 | 0.003 |
| 5 | 6 | 10 | 0.452 | 0.000 | 0.000 | 0.049 | 0.000 | 0.002 |
| 5 | 7 | 8 | 0.017 | 0.104 | 0.000 | 0.830 | 0.001 | 0.051 |
| 5 | 7 | 9 | 0.017 | 0.020 | 0.000 | 0.668 | 0.000 | 0.005 |
| 5 | 7 | 10 | 0.017 | 0.002 | 0.000 | 0.385 | 0.000 | 0.003 |
| 5 | 8 | 9 | 0.007 | 0.069 | 0.000 | 0.657 | 0.000 | 0.057 |
| 5 | 8 | 10 | 0.007 | 0.003 | 0.000 | 0.287 | 0.000 | 0.025 |
| 5 | 9 | 10 | 0.003 | 0.009 | 0.000 | 0.222 | 0.000 | 0.199 |
| 6 | 7 | 8 | 0.002 | 0.123 | 0.000 | 0.482 | 0.164 | 0.051 |
| 6 | 7 | 9 | 0.002 | 0.025 | 0.000 | 0.495 | 0.060 | 0.005 |
| 6 | 7 | 10 | 0.002 | 0.002 | 0.000 | 0.662 | 0.030 | 0.003 |
| 6 | 8 | 9 | 0.001 | 0.081 | 0.000 | 0.867 | 0.008 | 0.057 |
| 6 | 8 | 10 | 0.001 | 0.004 | 0.000 | 0.817 | 0.003 | 0.025 |
| 6 | 9 | 10 | 0.000 | 0.010 | 0.000 | 0.487 | 0.001 | 0.199 |
| 7 | 8 | 9 | 0.192 | 0.127 | 0.000 | 0.803 | 0.017 | 0.057 |
| 7 | 8 | 10 | 0.192 | 0.010 | 0.000 | 0.509 | 0.010 | 0.025 |
| 7 | 9 | 10 | 0.053 | 0.020 | 0.000 | 0.339 | 0.003 | 0.199 |
| 8 | 9 | 10 | 0.146 | 0.023 | 0.000 | 0.504 | 0.036 | 0.199 |

OS, overall survival; HR, hazard ratio; CI, confidence interval.
